# A Sensitive and Label-Free Pb(II) Fluorescence Sensor Based on a DNAzyme Controlled G-Quadruplex/Thioflavin T Conformation

**DOI:** 10.3390/s16122155

**Published:** 2016-12-16

**Authors:** Yanli Wen, Lele Wang, Lanying Li, Li Xu, Gang Liu

**Affiliations:** Biometrology Laboratory, Division of Chemistry and Ionizing Radiation Measurement Technology, Shanghai Institute of Measurement and Testing Technology, Shanghai 201203, China; wenyl@simt.com.cn (Y.W.); Wangll@simt.com.cn (L.W.); Lily@simt.com.cn (L.L.); Xuli@simt.com.cn (L.X.)

**Keywords:** DNAzyme, heavy metal ions, lead ion, thioflavin T (ThT)

## Abstract

Pb(II) can cause serious damaging effects to human health, and thus, the study of Pb^2+^ detection methods to sensitively and selectively monitor Pb(II) pollution has significant importance. In this work, we have developed a label-free fluorescence sensing strategy based on a Pb(II) DNAzyme cleavage and the ThT/G-quadruplex complex. In the presence of Pb(II), a G-rich tail was cut and released from the substrate strand, which then would form a G-quadruplex structure by combination with ThT dye. The fluorescence signal increase was then measured for sensitive Pb(II) quantification with a limit of detection of 0.06 nM. Our sensor also demonstrated high selectivity against six different metal ions, which is very important for the analysis of complex samples.

## 1. Introduction

Many heavy metal ions such as lead, mercury, arsenic and chromium are highly toxic elements which can cause a number of serious threats to human health and the environment [1,2,3,4,5]. Among all the metal ions, Pb^2+^ is one of the four metals that have the most damaging effects on human health. It can enter the human body through uptake of food, water and air, and cause serious health problems, including disruption of the biosynthesis of haemoglobin and anaemia, kidney damage, brain damage, diminished learning abilities of children and behavioral disruptions of children [4].

In order to protect human health from these threats, the study of sensitive, selective and low-cost Pb^2+^ detection methods to monitor lead ion pollution in many fields, including environmental, water, food safety, etc., has been of significant importance over the past decades. Recently, many different strategies have been developed toward efficient Pb^2+^ analysis, including classical atomic absorption spectrometry (AAS) [6,7,8], inductively coupled plasma mass spectrometry (ICP-MS) [9,10], and inductively coupled plasma atomic emission spectrometry (ICP-AES) [11,12]. Unfortunately, most of these methods require complicated sample pretreatment, multiple analysis steps or expensive equipment, hindering their application in on-site or real-time analysis.

Metal-specific DNAzymes are a class of well-characterized DNAzymes that cleave an oligonucleotide substrate containing one ribonucleotide at the cleavage site in the presence of a particular metal ion [13,14,15,16,17]. As oligonucleotides, high stability DNAzymes can be conveniently synthesized at low cost and DNAzyme-based sensors have demonstrated a series of advantages such as fast analysis, nondestructive detection and the capability of providing in situ and real-time information [18,19,20], and thus they have been widely recognized as promising candidates for the development of metal ion sensors. Recently, the research on Pb^2+^ sensors based on Pb^2+^-specific DNAzymes has attracted plenty of research interest. For instance, Lu’s group [3] reported a colorimetric Pb^2+^ biosensor based on the DNAzyme-directed assembly of gold nanoparticles. Xiao et al. [21] developed an electrochemical Pb^2+^ sensor via an electrode-bound DNAzyme assembly and achieved part-per-billion (nanomolar) sensitivity. Cropek’s group [22] reported a microchip-based lead sensor with a lead-specific DNAzyme and fluorescent tags which translated the cleavage events to measurable, optical signals proportional to Pb^2+^ concentration. However, most of the fluorescent Pb^2+^ biosensors need a covalently labeled fluorophore/quencher on the DNAzyme strand, often leading to complicated synthetic routes, high cost, low synthetic yield and even serious interference with DNAzyme cleavage [23,24]. Most recently, some groups have reported their inspiring progress in the development of label-free Pb^2+^ biosensors, yet the limit of detection (LOD) still remains a challenge for us [25,26,27,28].

Thioflavin T (ThT) is a commercial fluorescent dye, which is capable of binding to the G-quadruplex structure, generating an increased fluorescence signal, and it has been successfully utilized in several highly sensitive and label-free fluorescent biosensors [29,30,31,32]. In this work, we designed a novel DNAzyme analysis system by tailing a G-rich sequence onto the substrate strand. In the presence of Pb^2+^, DNAzyme cleaved the substrate strand and released the G-rich part which subsequently combined with ThT and formed a G-quadruplex structure for an obviously enhanced fluorescent signal. Our strategy achieved excellent selectivity of Pb^2+^ over six different metal ions, and the limit of detection (LOD) of Pb^2+^ was 0.06 nM and the linear range was from 10 nM to 10 μM Pb^2+^.

## 2. Materials and Methods

### 2.1. Materials

Pb^2+^-specific DNAzyme (Pb-DNAzyme) and the substrate strand DNA (Pb-sub) were synthesized and purified by TaKaRa Biotech. Co., Ltd. (Dalian, China), and the DNA sequences are shown as follows:

Pb-DNAzyme:
  5′-CCAAAGTGCTCCGAGCCGGTCGAAGTGAAACC-3′
Pb-Sub:
  5′-GGGTTGGGCGGGATGGGTTTCACTrAGGCACTTTGGGTAGGG-3′, (rA represents an adenosine ribonucleotide).

Pb(NO_3_)_2_, Cu(NO_3_)_2_, Cd(NO_3_)_2_, Co(NO_3_)_2_, Mn(NO_3_)_2_, Ni(NO_3_)_2_, Hg(NO_3_)_2_ were 1 g/L certified reference materials (CRMs) obtained from the Shanghai Institute of Measurement and Testing Technology (Shanghai, China). 4-(2-Hydroxyethyl)-1-piperazineethane sulfonic acid (HEPES) and 3,6-dimethyl-2-(4-dimethylaminophenyl) benzothiazolium cation (ThT) were purchased from Sigma-Aldrich (St. Louis, MO, USA). All reagents were of analytical grade and all solutions were prepared using ultrapure water (18.2 MΩ·cm resistivity).

### 2.2. Apparatus

Fluorescence spectra were measured on a F-7000 spectrophotometer (Hitachi, Tokyo, Japan) equipped with a 150 W xenon lamp excitation source, using a quartz cell of 10 mm path length. The excitation wavelength was 425 nm, and the emission spectrum was from 450 nm to 600 nm. The slits for excitation and emission were 2.5 nm. A nanodrop 2000 spectrophotometer (Thermo Scientific, Waltham, MA, USA) was used to quantify the oligonucleotides by measuring the UV absorbance at 260 nm (OD260).

### 2.3. DNAzyme-Based Fluorescence Sensor for Pb^2+^

Pb^2+^ solutions were prepared in ultrapure water of a series of concentrations, and then 0.3 μM Pb-DNAzyme, 0.2 μM Pb-sub DNA, 50 mM KCl and 10 mM HEPES buffer were added, and then the mixture was incubated in 37 °C air bath for 2 h to firstly form the substrate-enzyme complex DNAzyme and then perform the DNAzyme cleavage. After the cleavage, 20 μM ThT was added to react with the G-rich sequence at 37 °C for 30 min to form the ThT/G-quadruplex complex. Finally, the fluorescence signal at 490 nm was measured.

## 3. Results

### 3.1. The Principle of Pb^2+^ Detection

**Figure 1:**
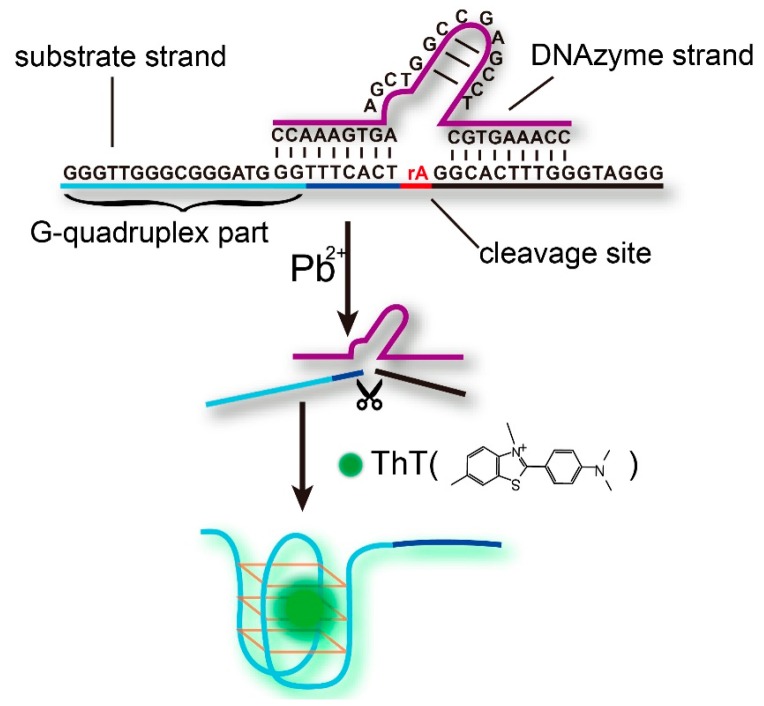
Illustration of the label-free fluorescent sensing strategy based on the Pb^2+^-DNAzyme and ThT/G-quadruplex complex.

The label-free fluorescent strategy for Pb^2+^ sensing based on the DNAzyme and the ThT/G-quadruplex complex is shown schematically in Figure 1. The sensing system consists of a DNAzyme strand (Pb-DNAzyme), a substrate strand (Pb-sub), and a single, ribo-adenine (rA, indicated as a red point in Figure 1) between them. The substrate strand is tailed with a G-rich sequence at the 5′ end, and most importantly, part of the G-rich sequence hybridized to the DNAzyme strand, inhibiting the formation of G-quadruplex structure. In the presence of Pb^2+^ ions, Pb-sub is cleaved at the rA site, and thus released the G-rich strand to form a G-quadruplex structure under the combination of ThT dye. Finally, an obviously enhanced fluorescence signal was obtained, and the increase in the fluorescence intensity was calculated as the signal gain for Pb^2+^ quantification through F-F_0_, where F_0_ and F are the fluorescence intensity before and after Pb^2+^ DNAzyme reaction, respectively. CD Spectrum (Figure S1) and polyacrylamidegel electrophoresis results (Figure S3) demonstrated the formation of G-quadruplex, and when we increased the time of DNAzyme cleavage, the signal gain (F-F_0_) increased until it reached a plateau at 2 h (Figure S2).

### 3.2. Optimization of the Detection Conditions

**Figure 2:**
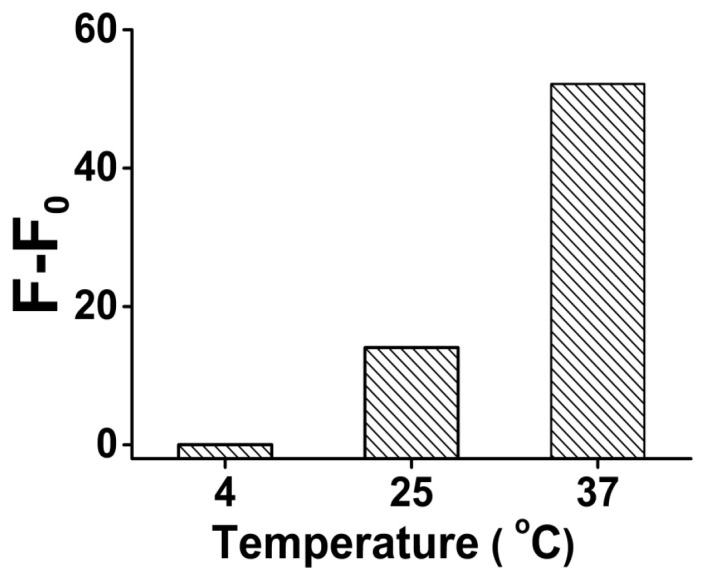
The optimization result of the analysis temperature for Pb^2+^ sensing in the presence of 1 μM Pb^2+^. Original data for the optimization result of the analysis temperature shown in Figure S4.

As a DNAzyme-based analysis, the reaction temperature is a key characteristic in the system. Our results showed that when we raised the reaction temperature from 4 °C to 37 °C, the signal gain (F-F_0_) increased more than 2000 times (from 0.02 to 52.17) (Figure 2), because the higher temperature improved the DNAzyme activity, and promoted the reaction kinetics of the liquid phase reaction system. Meanwhile, the unspecific DNA secondary structure was eliminated under higher temperature to suppress the background noise.

**Figure 3:**
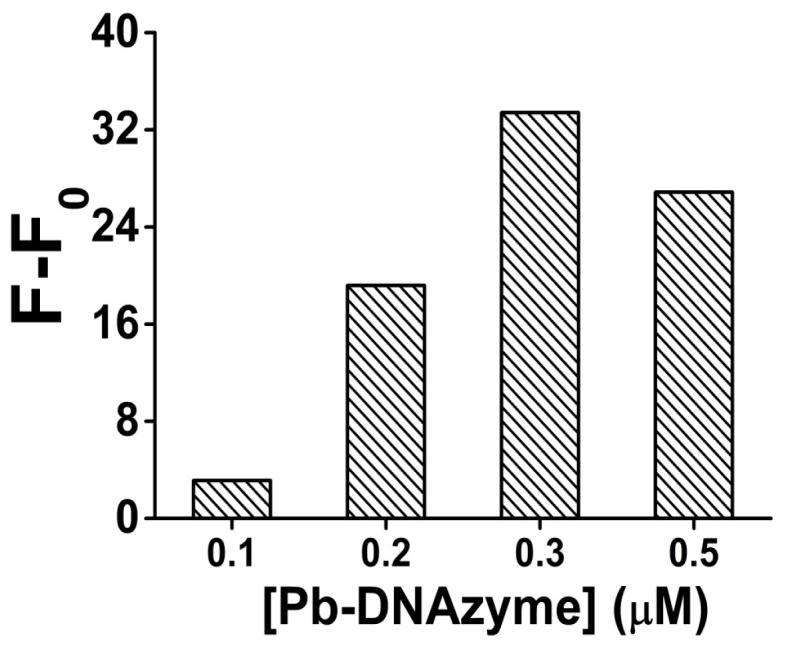
The optimization result of the Pb-DNAzyme concentration for Pb^2+^ sensing in the presence of 100 nM Pb^2+^. [Pb-DNAzyme] is the concentration of Pb-DNAzyme. Original data for the optimization result of the Pb-DNAzyme concentration for Pb^2+^ sensing shown in Figure S5.

Next, in order to verify the influence of DNAzyme amount, the cleavage reaction was performed with different DNAzyme final concentrations. After 2 h incubation with and without Pb^2+^ (100 nM), the fluorescence signal gain was compared (Figure 3). As the results show, the signal gain obviously increased as the concentration of Pb-DNAzyme increased from 0.1 to 0.3 μM, because more DNAzyme provided higher cleavage efficiency, generating a higher signal gain, However, when the concentration of Pb-DNAzyme exceeded 0.3 μM, the signal gain decreased slightly, maybe because too much background fluorescent signal was produced due to unspecific cleavage, even without the combination with Pb^2+^. As a result, we chose 0.3 μM as the optimized Pb-DNAzyme concentration in our analysis.

ThT dye was very important for the fluorescence signal generation, through recognizing and intercalating into the G-quadruplex structure after the G-rich sequence was cleaved by DNAzyme from the Pb-sub strand, although, like with many other fluorescent DNA intercalators, if too much dye is added, a higher background fluorescence signal might happen because of its unspecific binding to the double strand DNA or other DNA second structures. In order to improve the signal-to-noise ratio, we investigated the relationship between ThT concentration and the fluorescence signal, while all other experiment conditions were fixed including the final concentration of Pb-sub strand (0.2 μM) and Pb-DNAzyme strand (0.3 μM).

**Figure 4:**
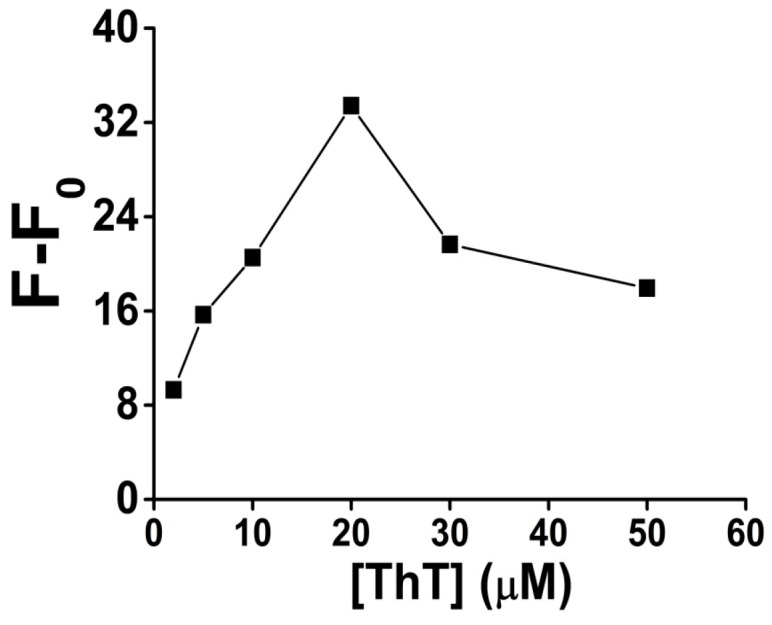
The optimization result of ThT concentration for Pb^2+^ sensing in the presence of 100 nM Pb^2+^. [ThT] is the concentration of ThT. Original data for the optimization result of ThT concentration for Pb^2+^ sensing shown in Figure S6.

As shown in Figure 4, the fluorescence signal gain increased obviously as the ThT concentration increased from 2 to 20 μM. However, when the concentration of ThT is higher than 20 μM, the fluorescence signal gain decreased, mainly due to the increase of the fluorescent background. Thus, 20 μM ThT was used as an optimized condition in all our following analysis.

### 3.3. Quantification of Pb^2+^

**Figure 5:**
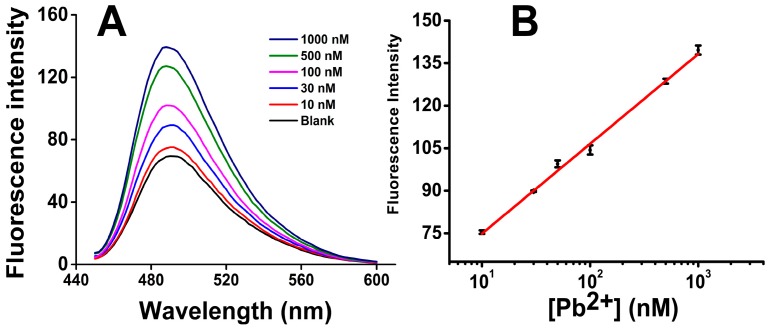
Quantification results of Pb^2+^: (**A**) The fluorescence spectra of ThT/G-quadruplex in the presence of Pb^2+^ from 10 to 1000 nM; (**B**) Plot for the concentration of Pb^2+^ vs. fluorescence signal gain. Error bars show the standard deviations of measurements taken from independent experiments with at least three distinct sensors.

In order to investigate the sensitivity, a series of Pb^2+^ solutions were prepared with different concentrations and then analyzed by our Pb^2+^ sensor. Figure 5A shows the fluorescence spectra of ThT/G-quadruplex in the presence of Pb^2+^ from 10 to 1000 nM. As the concentration of Pb^2+^ increased, the fluorescence intensity increased obviously, indicating successful DNAzyme cleavage of the Pb-Fl/Pb-Sub duplex and the formation of ThT/G-quadruplex structure.

As Figure 5B shows, the fluorescence signal gain has a good linear relationship with the base 10 log of Pb^2+^ concentration from 10 nM to 1000 nM: F = 31.6857lg[Pb^2+^] − 43.1832, where the [Pb^2+^] is the concentration of Pb^2+^. The background signal F_0_ was 69.91 ± 1.23 when a blank sample was analyzed. The limit of detection (LOD) was thus calculated to be 0.06 nM, using the 3δ (3 × 1.23) in the fitting equation.

### 3.4. Specificity of Pb^2+^ Analysis

**Figure 6:**
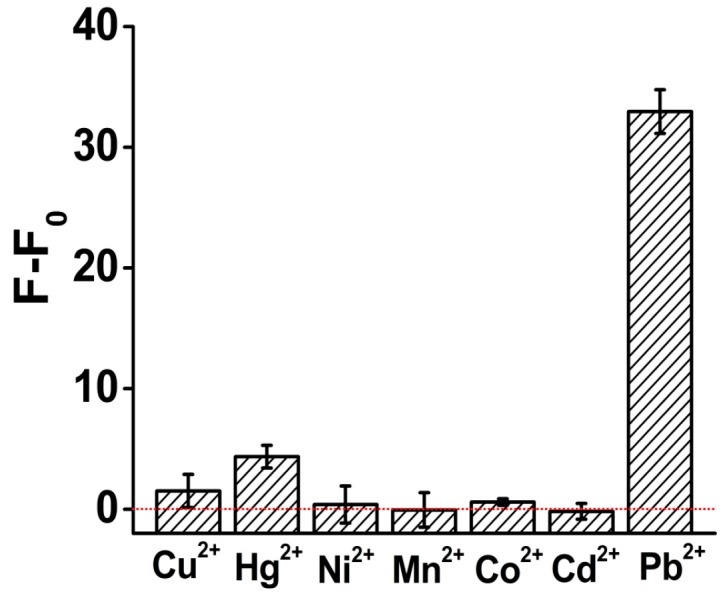
Investigation results of the specificity of the Pb^2+^ sensor. The concentration of all the metal ion solutions was 100 nM. All the analysis experiments were performed under the same condition.

To challenge the specificity for the Pb^2+^ sensor, we then investigated the response of ThT/G-quadruplex towards several other divalent metal ions including Cu^2+^, Hg^2+^, Co^2+^, Ni^2+^, Cd^2+^ and Mn^2+^ under the same analysis condition. As shown in Figure 6, our sensor generated a significant fluorescence enhancement for 100 nM Pb^2+^, which is 8~100 times higher than for the other ions, and the signal gain for some of the interfering ions was only negligible. Our results thus demonstrated that our sensing system had a high selectivity to Pb^2+^ over other metal ions.

Our DNAzyme-based sensor represents a new progress in the family of label-free Pb^2+^ sensors. It is an easy-to-operate sensor with 0.06 nM sensitivity that exceeds most of the previously reported label-free Pb^2+^ sensors by several orders of magnitude (Table 1). We also note that this sensor showed a wider and practical linear range from 10 nM to 1 μM.

## 4. Conclusions

In this work, we constructed a novel Pb^2+^ fluorescence sensor, based on a DNAzyme directed G-quadruplex/ThT conformation. Our results demonstrated the excellent sensing performance of the DNAzyme when Pb^2+^ was added. The sensitivity was 0.06 nM which is much lower than EPA-defined maximal contamination level for Pb^2+^ in drinking water (72 nM) and is, to our knowledge, quite competitive among all the reported label-free Pb^2+^ sensors. The analysis was conveniently performed in one tube in two simple steps, avoiding unnecessary contamination and oxidation, thus our quantification results were so stable that the relative standard deviations (RSD) were all below 5% except for only the RSD for 10 nM Pb^2+^ (11%). Importantly, good selectivity was achieved compared with six different metal ions, indicating a great potential application of our sensor for assays in complex samples.

## Figures and Tables

**Table 1:** Comparison of similar Pb^2+^ sensors.

DNA Probe	Linear Range	Detection Limit	Detection Methods	Detection Time	Reaction System	Reference
G-quadruplex	1 ng/mL –1 mg/mL, R^2^ = 0.992	1 ng/mL (4.83 nM)	Fluorescence	0.5 h	DsDNA’s conformational changes/PicoGreen	[25]
Allosteric G-quadruplex DNAzyme	1 nM –316 nM, R^2^ = 0.980	1 nM	Chemiluminescence	2 h	luminol-H_2_O_2_	[26]
DNAzymes/AUR	0 nM–1000 nM, R^2^ = 0.95	0.4 nM	Fluorescence	3.5 h	G4/hemin/H_2_O_2_/AUR catalytic system	[27]
G-quadruplex DNAzyme	5 nM–100 nM, R^2^ = 0.996	3 nM	Fluorescence	3.5 h	catalytic beacons/G4/ZnPPIX	[28]
G-quadruplex DNAzyme	10 nM–1000 nM, R^2^ = 0.997	0.06 nM	Fluorescence	2.5 h	G4/ThT	This work

## Supplementary Materials

The following are available online at http://www.mdpi.com/1424-8220/16/12/2155/s1. Figure S1. CD spectrum of different samples. Figure S2. A time-dependent amplification of the fluorescence sensor for 50 nM Pb^2+^ detection. Figure S3. Polyacrylamidegel electrophoresis results of different samples. Figure S4. Original data for the optimization result of the analysis temperature (A) 4 °C; (B) 25 °C; (C) 37 °C in the presence of 1 μM Pb^2+^. Figure S5. Original data of 100 nM Pb^2+^ sensing in the presence of different concentration of Pb-DNAzyme: (A) 0.1 μM; (B) 0.2 μM; (C) 0.3 μM; (D) 0.5 μM. Figure S6. Original data of 100 nM Pb^2+^ sensing using different concentration of ThT: (A) 2 μM; (B) 5 μM; (C) 10 μM; (D) 20 μM; (E) 30 μM; (F) 50 μM.
