# Supplementary material for: A Sensitive and Label-Free Pb(II) Fluorescence Sensor Based on a DNAzyme Controlled G-Quadruplex/Thioflavin T Conformation
Source: Sensors (Basel). 2016 Dec 16;16(12):2155. doi: 10.3390/s16122155 (PMC5191135; doi:10.3390/s16122155)
Supplement: Supplementary file 1 [file sensors-16-02155-s001.pdf]

Supplementary Materials: Sensitive and Label-Free Pb(II) Fluorescent Sensor Based on DNAzyme Controlled G-Quadruplex/Thioflavin T Conformation

Yanli Wen, Lele Wang, Lanying Li, Li Xu and Gang Liu

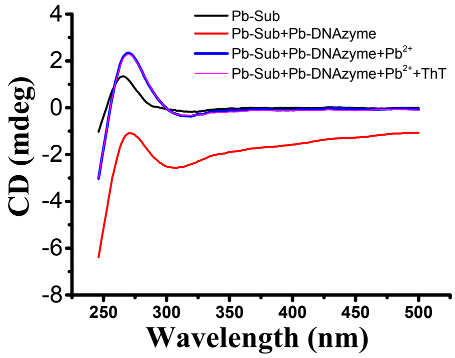

Figure S1. CD spectrum of different samples.

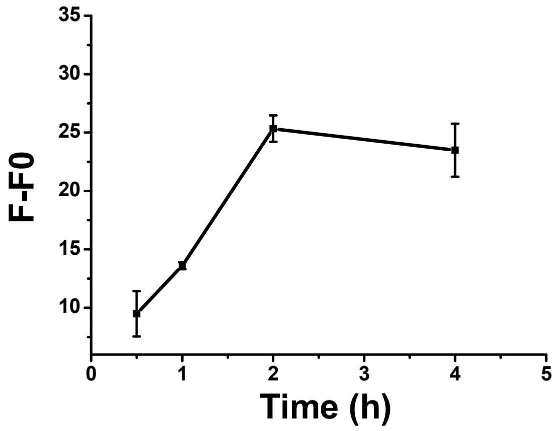

Figure S2. A time-dependent amplification of the fluorescence sensor for 50 nM Pb^{2+} detection.

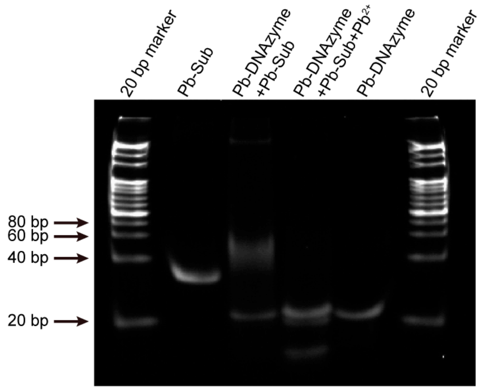

Figure S3. Polyacrylamidegel electrophoresis results of different samples.

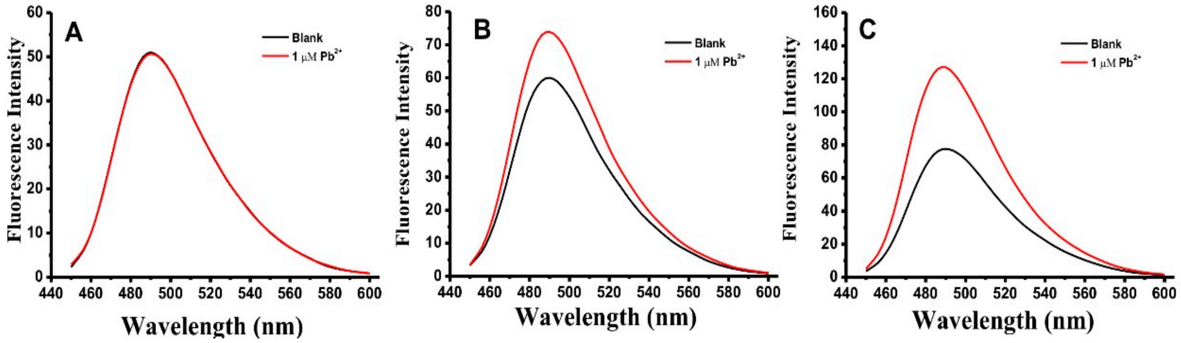

Figure S4. Original data for the optimization result of the analysis temperature (**A**) 4 °C; (**B**) 25 °C; (**C**) 37 °C in the presence of 1 μM Pb^{2+}.

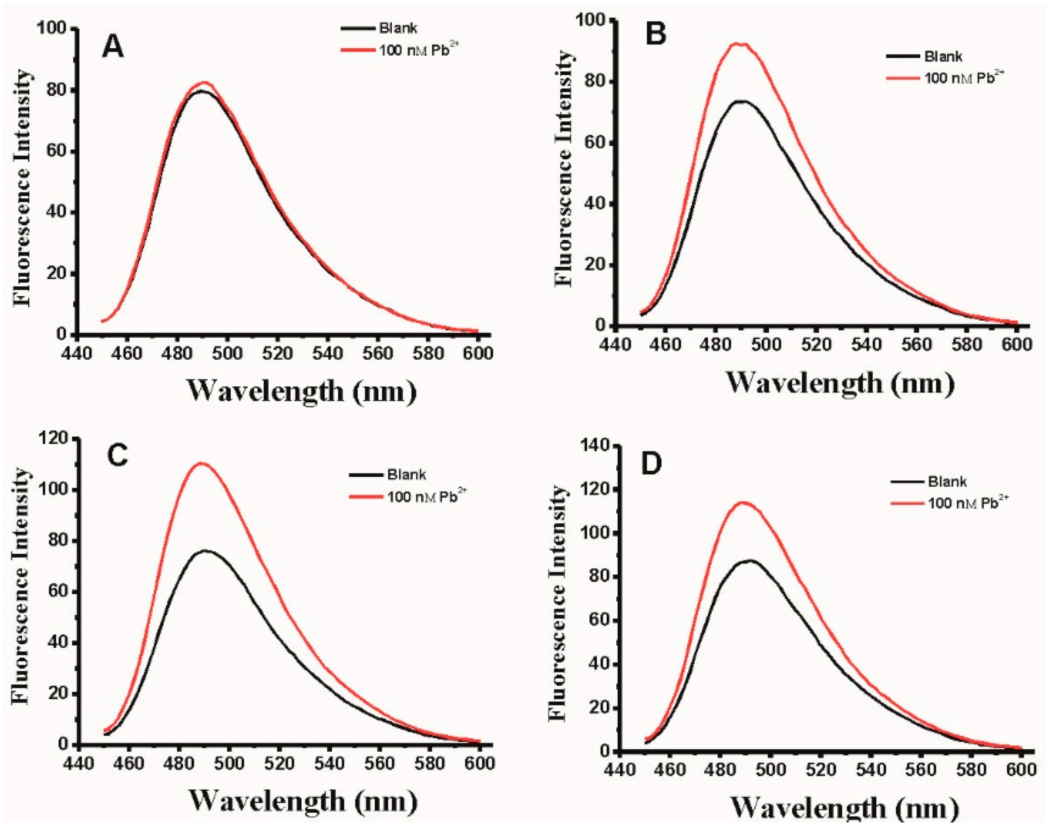

Figure S5. Original data of 100 nM Pb^{2+} sensing in the presence of different concentration of Pb-DNAzyme: (**A**) 0.1 μM; (**B**) 0.2 μM; (**C**) 0.3 μM; (**D**) 0.5 μM.

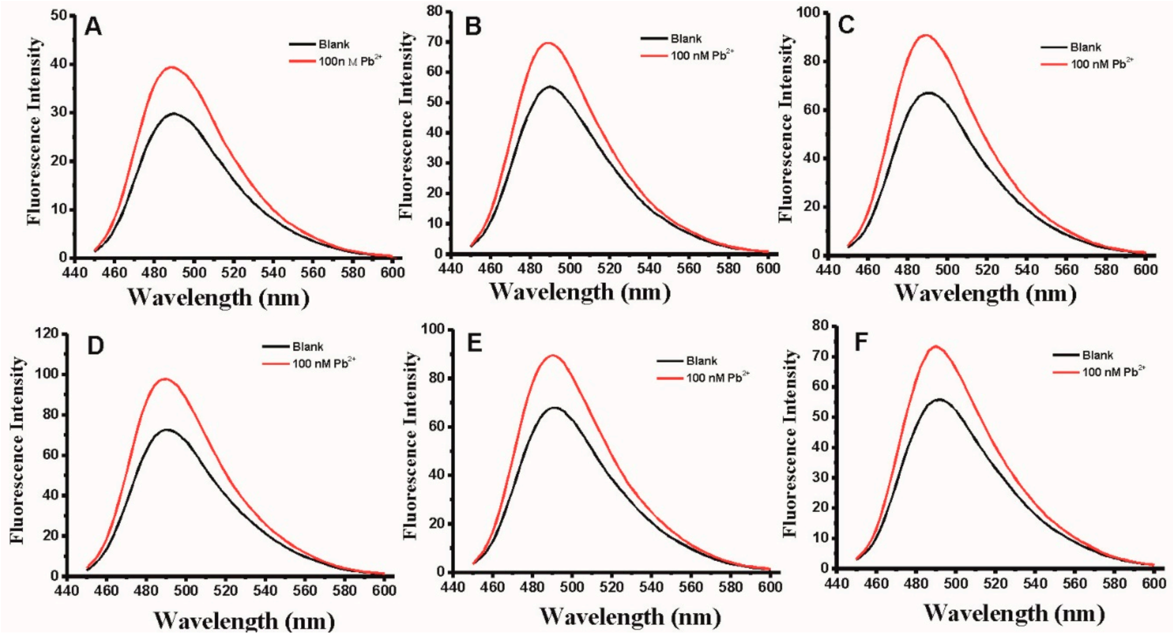

Figure S6. Original data of 100 nM Pb^{2+} sensing using different concentration of ThT: (**A**) 2 μM; (**B**) 5 μM; (**C**) 10 μM; (**D**) 20 μM; (**E**) 30 μM; (**F**) 50 μM.
